# Anesthetic management using a combination of anterior quadratus lumborum block and erector spinae plane block for robot-assisted partial nephrectomy: two case reports

**DOI:** 10.1186/s40981-020-00371-2

**Published:** 2020-08-19

**Authors:** Nobuhiro Tanaka, Takuzo Kitazawa, Saki Mitani, Takanori Suzuka, Yuma Kadoya, Masahiko Kawaguchi

**Affiliations:** Department of Anesthesiology, Nara Medical University, 840 Shijo-cho, Kashihara, Nara, 634-8522 Japan

**Keywords:** Erector spinae plane block, Peripheral nerve block, Quadratus lumborum block, Robot-assisted partial nephrectomy

## Abstract

**Background:**

There has been increasing attention regarding quadratus lumborum block (QLB) and erector spinae plane block (ESPB) as effective truncal blocks. There have been reports of combined QLB and ESPB usage in hip surgery resulting in a symbiotic increase in effectiveness. However, there have been no reports regarding robot-assisted partial nephrectomy (RAPN), which requires multiple port holes ranging from near the xiphoid process to below the umbilicus. We hypothesized that the combined use of QLB and ESPB was an option for anesthesia and analgesia during RAPN.

**Case presentation:**

Anterior QLB and ESPB were applied to two patients undergoing scheduled RAPN. With intravenous patient-controlled analgesia, the post-surgery numerical rating scale scores were < 3/10 at rest and < 5/10 upon movement, throughout the perioperative time.

**Conclusions:**

The combination of QLB and ESPB could be an option for the postoperative analgesia in RAPN.

## Background

Based on increasing studies regarding the mechanism and efficacy of quadratus lumborum block (QLB), QLB is considered suitable for lower abdominal surgery and hip surgery [1–3]. However, attention should be paid to the indications for abdominal surgery since Tamura et al. suggested that QLB (posterior and intramuscular) efficacy may be limited to the lateral abdominal region [4, 5]. Specifically, QLB may not be indicated for abdominal surgery with a midline incision; however, its effect on flank surgery remains unclear. Specifically, anterior QLB (including subcostal anterior QLB) has been reported to be effective in post-nephrectomy anesthesia and postoperative analgesia [6, 7]. These studies indicate that the sensory loss area is from Th6–L2 at maximum; however, the stability involved is unclear.

A few case reports have shown that erector spinae plane block (ESPB) is an effective block during nephrectomy [8, 9]. In these block procedures, local anesthetic diffusion is an important effect-determining factor.

**Fig. 1:**
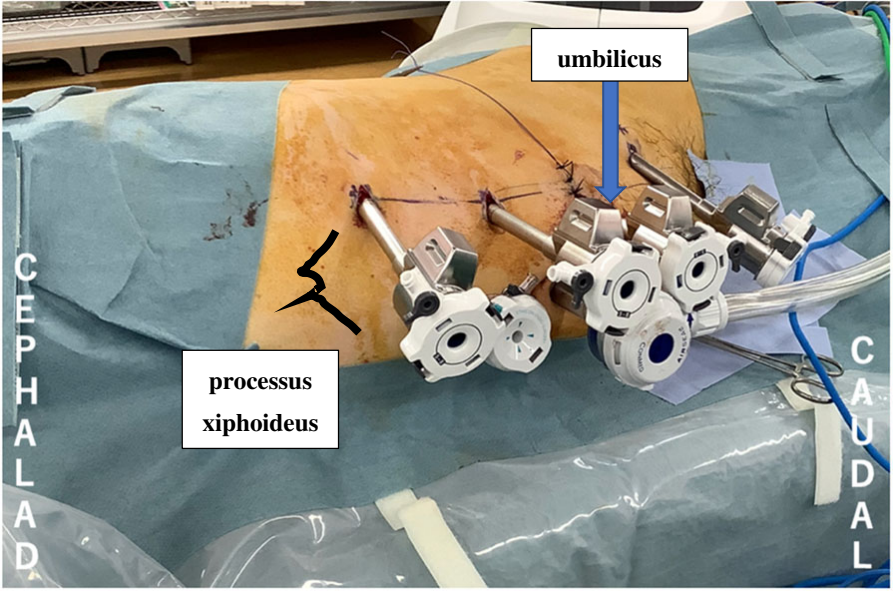
Port placement for robot-assisted laparoscopic partial nephrectomy in the right decubitus position (case 1). The port holes are widely distributed from near the xiphoid process to the umbilicus

We hypothesized that combining QLB and ESPB could achieve the effect with a higher probability and wider range by devising the puncture site. Robot-assisted partial nephrectomy (RAPN) requires multiple port holes ranging from near the xiphoid process to below the umbilicus (Fig. 1) [10]. Therefore, we expected the QLB to control visceral and somatic pain below the Th10 level and the ESPB to control above the Th10 level.

## Case presentation

We obtained written informed consent from the patients for the combined usage of QLB and ESPB and for publication of this report.

### Case 1

**Fig. 2:**
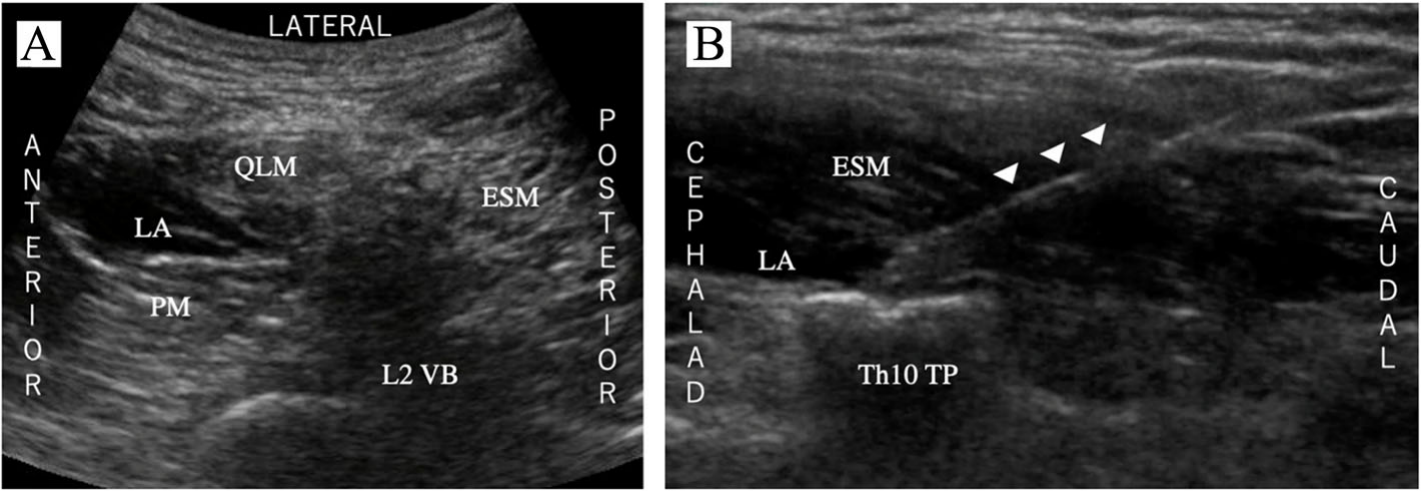
Ultrasound image of local anesthetic (LA) spread. **a** The anterior QLB. LA spread between the quadratus lumborum muscle and psoas major muscle (PM). **b** The ESPB. The needle (arrowhead) placed at the top of the transverse process of Th10. LA spread below the erector spinae muscle. ESM, erector spinae muscle; LA, local anesthetic; QLM, quadratus lumborum muscle; PM, psoas major muscle; TP, transverse process; VB, vertebral body

A 71-year-old male patient (174 cm, 56 kg) with a 15-mm large mass in the upper pole of the left kidney was scheduled for the RAPN peritoneal approach. Rapid induction was achieved using fentanyl, propofol, and rocuronium, which were added after establishing peripheral intravenous access. Anesthesia was maintained using desflurane and remifentanil. After tracheal intubation, and assuming the right decubitus position, the anterior QLB and ESPB were performed (Fig. 2). A convex probe (2–5 Hz) was placed on the L2 vertebral body, and we attempted to obtain the shamrock view, which overlooks the quadratus lumborum muscle (QLM), erector spinae muscle (ESM), psoas major muscle (PMM), and vertebral body. A 100-mm block needle (20-G Tuohy needle) was inserted using the in-plane technique; moreover, 30 mL of 0.25% ropivacaine was applied between the QLM and PMM as the anterior QLB. Subsequently, the ESPB was performed at the transverse process of Th10. From this point, the needle was inserted in the caudal-to-cephalad direction. After confirming that the needle reached the ESM, we injected 30 mL of 0.25% ropivacaine. The operation was performed via the peritoneal approach with six port holes. The operative and anesthetic times were 209 and 294 min, respectively.

The patient intraoperatively received intravenous fentanyl 300 μg and acetaminophen 1000 mg. Postoperative pain was managed in the ward using continuous intravenous infusion of 15 μg/h fentanyl combined with 15 mcg fentanyl for intravenous patient-controlled analgesia (IV-PCA) with a 10-min lockout.

A pinprick test was performed 2 h post-surgery using the Semmes-Weinstein monofilament 2.0 g (SAKAI Medical Co., Tokyo, Japan) as part of the postoperative procedures by someone who did not perform the blocks. The pinprick test revealed that the anesthetized dermatomal range was Th7–Th12. However, tactile sensation was normal around the port site in the midline of the umbilicus. The numerical rating scale (NRS) was 0-0-0-0/10 at rest and 1-1-1-5/10 on movement at 2, 12, 24, and 48 h after surgery, respectively. IV-PCA was stopped at 20 h after surgery due to postoperative nausea and low NRS value. The total fentanyl amount postoperatively used was 450 μg. Acetaminophen was administered once at postoperative day (POD) 1. He was discharged without any adverse events at POD 6.

### Case 2

A 68-year-old female patient (161 cm, 50 kg) with a 21-mm large mass in the upper pole of the right kidney was scheduled for RAPN peritoneal approach. The anesthetic method and block were similar to those in case 1.

The operative and anesthetic times were 271 and 326 min, respectively. The patient intraoperatively received intravenous fentanyl 200 μg and acetaminophen 750 mg. The surgical procedure was different from that of case 1 with respect to the placement of a single and surgery time extension. Postoperative pain was managed in the ward using continuous intravenous infusion of 25 μg/h fentanyl combined with 25 μg fentanyl for IV-PCA with 10-min lockout.

A pinprick test performed at 2 h after surgery revealed that the anesthetized dermatomal range was Th7–Th12. The NRS scores were 3-0-0-0/10 at rest and 5-1-1-3/10 on movement at 2, 12, 24, and 48 h after surgery, respectively. Acetaminophen was administered once at POD 0. IV-PCA was stopped at 67 post-surgery hours. The total fentanyl amount post-operatively used was 2425 μg. She was discharged without adverse events at 7 days after surgery.

## Discussion and conclusions

RAPN is a minimally invasive option for patients with small renal masses undergoing partial nephrectomy. However, it requires multiple port holes that widely range from near the xiphoid process to below the umbilicus. Jin et al. reported no significant difference in postoperative pain between patients who underwent RAPN and laparoscopic partial nephrectomy. The NRS scores in the RAPN group anesthetized by general anesthesia only were 5.9, 3.5, and 2.8 at POD 0, 1, and 2, respectively [11].

Although epidural anesthesia could provide reliable pain relief, it has several side effects, including paresthesia, hypotension, urinary disturbance, and epidural hematoma, which increase the risk of anticoagulant therapy in the early postoperative period.

QLB are classified into three main types: posterior, lateral, and anterior approach [12]. The anterior QLB has been reported to have great efficacy in hip surgery and lower abdominal surgery [1–3]. Recent studies have shown that the anterior QLB is suitable for flank surgery rather than abdominal surgery with a midline incision [4, 5, 13]. The anterior QLB, especially the subcostal anterior QLB, have been suggested to be effective in nephrectomy [6, 7]. We chose the anterior QLB at L2 level, which we are familiar with, rather than the subcostal anterior QLB. Moreover, the anterior QLB at L2 level had a wide anesthetized range but a variable pattern [13].

ESPB has quickly become a popular technique for thoracic, abdominal, and extremity surgeries since its first report in 2016 [14]. The efficacy of ESPB is considered to involve both the ventral and dorsal rami of the spinal nerves; however, the exact pathway of local anesthetic diffusion remains unclear.

We hypothesized that the combined use of QLB and ESPB could increase the probability and range of the effect by devising the puncture site. A previous case report on the combined use of ESPB and QLB for hip surgery highlighted the importance of their mutual increase in effectiveness and complementing each other’s missing aspects [15]. In the present cases, both patients weighed over 50 kg. Therefore, the maximum dose of ropivacaine (3 mg/kg) was 150 mg. In QLB, it is believed that the analgesia is due, in part, to the local anesthetic (LA) spread along the thoracolumbar and endothoracic fascia into the paravertebral space. In ESPB, LA diffuses anteriorly to the ventral and dorsal rami of the spinal nerves and through the intertransverse connective tissue to enter the paravertebral space due to the discontinuity of the intercostal muscles. According to previous reports, these fascial plane blocks rely on a high-volume, low-concentration technique for optimal efficacy. We therefore decided to use as much local anesthetics as possible within the range currently reported and applied 0.25% ropivacaine 30 mL [16, 17].

The postoperative pain evaluation with NRS was performed at 2 h, 24 h, and 48 h after surgery. There was an increase in NRS on movement between 24 and 48 h after surgery despite the same condition for IV-PCA and postoperative rehabilitation. From these results, we considered that the combination of QLB and ESPB was effective for more than 24 h, but less than 48 h, after surgery. As the single-shot technique was used in these cases, there is a need for additional studies to assess nerve block strategies using catheter placement for better analgesia. This is because the NRS score was 5 on movement at 48 h after surgery in case 1, which suggests that a single injection resulted in recurrent pain.

Each anesthesiologist determined the dose of fentanyl for IV-PCA. Fentanyl usage was large in case 2. The mechanism underlying postoperative pain in partial nephrectomy is considered to involve port pain, small incisions for tumor extraction, pelvic organ nociception, diaphragmatic irritation, ureteric colic, and urinary catheter discomfort [18]. Although the effect range of QLB and ESPB appears appropriate in case 2, the patient presented with visceral pain, which was attributed to the placement of a single J ureteral stent that was removed at POD 4. Generally, 80% of patients with indwelling urinary stents feel uncomfortable and often complain of flank pain [19, 20]. We recognized that the continued use of IV-PCA during single J stent placement resulted in increased fentanyl usage in case 2. Despite the facts mentioned above, good analgesia was obtained under the combination of QLB and ESPB with IV-PCA connected.

To our knowledge, this is the first report of the combined use of ESPB and anterior QLB for RAPN. These cases indicate that the combined use of ESPB and anterior QLB is an effective postoperative analgesia strategy in RAPN.

## Data Availability

Data relevant to this case report are unavailable for public access because of patient privacy concerns.

## Abbreviations

ESM: Erector spinae muscle
ESPB: Erector spinae plane block
IV-PCA: Intravenous patient-controlled analgesia
LA: Local anesthetic
NRS: Numerical rating scale
PMM: Psoas major muscle
POD: Postoperative day
QLB: Quadratus lumborum block
QLM: Quadratus lumborum muscle
RAPN: Robot-assisted partial nephrectomy
